# Emergence of Novel Reassortant H3N2 Avian Influenza Viruses in Southern China: Genetic Complexity and Pathogenicity in Chickens and Mice

**DOI:** 10.3390/ani16121765

**Published:** 2026-06-08

**Authors:** Meichi Chen, Yanjiao Liang, Changmao Jian, Changting Li, Junwei Yang, Jingting Yang, Kewei Chen, Miaoxiang Zhang, Meilan Mo, Tianchao Wei, Teng Huang, Jianni Huang

**Affiliations:** 1 College of Animal Science and Technology, Guangxi University, Nanning 530004, China; 17381092659@163.com (M.C.); liangyanjiao1219@163.com (Y.L.); 15120390804@163.com (C.J.); 17655993115@163.com (J.Y.); yjt15650122411@163.com (J.Y.); ckw286513@163.com (K.C.); chouchoulizmx@163.com (M.Z.); momeilan@gxu.edu.cn (M.M.); tcwei88@126.com (T.W.); tenhhwang@163.com (T.H.); 2 Guangxi Key Laboratory of Veterinary Biotechnology, Guangxi Veterinary Research Institute, Nanning 530001, China; lctyq0508@163.com; 3 Guangxi Key Laboratory of Animal Breeding, Disease Control and Prevention, Nanning 530004, China; 4 Guangxi Zhuang Autonomous Region Engineering Research Center of Veterinary Biologics, Nanning 530004, China

**Keywords:** avian influenza virus, H3N2 subtype, genetic diversity, pathogenicity, transmission

## Abstract

H3N2 subtype avian influenza virus (AIV) is prevalent in poultry and wild birds and typically causes asymptomatic or mild respiratory infections. However, genetic reassortment between H3N2 and other AIV subtypes generates novel strains capable of crossing the species barrier, posing a threat to both poultry and public health. In this study, nine H3N2 AIVs were isolated from ducks in live poultry markets (LPMs) in Guangxi, southern China, during 2022–2024. Phylogenetic analysis revealed that all eight gene segments of the nine isolates were clustered within the Eurasian lineage, with internal genes derived from multiple subtypes, including H1, H2, H3, H4, H5, H6, H7, and H9. These findings indicate complex gene reassortment of H3N2 AIVs in Guangxi. Importantly, the PB2 genes of certain isolates were closely related to those of highly pathogenic H5 subtype viruses, suggesting that H3N2 AIVs may contribute internal genes to H5 viruses. Three representative isolates (LZD44, NND98, and NND100) were assessed for pathogenicity in SPF chickens and mice. All three strains successfully replicated in the respiratory tissues of both species. Notably, the LZD44 virus, which harbored the mammalian-adaptive mutations PB2-MVV and NP-I353V, presented significantly higher virulence in chickens and mice than the other two strains. These results demonstrate that H3N2 subtype AIVs are capable of replicating in certain tissues of chickens and mice without prior adaptation, underscoring a potential risk for cross-species transmission. Consequently, sustained surveillance of H3N2 subtype AIVs is essential to prevent the spillover of novel recombinants into the human population.

## Simple Summary

H3N2 avian influenza virus (AIV) is prevalent in poultry and wild birds; however, certain strains possess the potential to evolve and cross the species barrier, thus posing a significant threat to public health. This study investigated the genetic evolution and pathogenicity of H3N2 subtype AIVs in southern China. The results showed that all eight gene segments of the nine isolates were clustered within the Eurasian lineage, with internal genes derived from multiple subtypes (including H1, H2, H3, H4, H5, H6, H7, and H9), indicating complex gene reassortment of H3N2 AIVs. Three representative isolates (LZD44, NND98, and NND100) replicated in various tissues of chickens and mice. Significantly, the LZD44 virus, which harbored the mammalian-adaptive mutations PB2-MVV and NP-I353V, exhibited higher virulence in chickens and mice than the other two strains. These findings indicate that H3N2 AIVs possess the capability to transmit to other species without prior adaptation, highlighting a risk of interspecies transmission. Therefore, continuous monitoring of H3N2 subtype AIVs is crucial for the effective prevention and management of avian influenza.

## 1. Introduction

Avian influenza viruses (AIVs), which belong to the family Orthomyxoviridae, are responsible for respiratory, gastrointestinal, and reproductive diseases in infected birds. On the basis of their pathogenicity in chickens, AIVs are classified into highly pathogenic viruses (HPAIVs) and low-pathogenicity avian influenza viruses (LPAIVs) [1]. H3 subtype AIVs, which are LPAIVs, circulate widely in wild birds and poultry [2,3,4]. However, the H3N2 influenza virus linked to the 1968 influenza pandemic emerged from reassortment between avian H3N2 and human H2N2 viruses, with avian-derived HA and PB1 gene segments contributing to the recombinant virus [5,6]. The gene segments of H3 AIVs isolated from Asian swine in 1977 were derived from duck-origin H3N2 strains [7]. Additionally, three canine influenza viruses, which originate from avian H3N2 AIVs, have been isolated from dogs with severe respiratory disease [8]. Therefore, the evolutionary dynamics of H3 subtype viruses and their capacity for cross-species transmission pose significant risks to both animal and human health.

Based on the hemagglutinin (HA) long-term evolution of the H3 subtype, the Eurasian and North American lineages are recognized phylogenetically [2,3,9]. The Eurasian lineage is characterized by a broad host range, including swine, equine, canine, and human. In contrast, viruses of the North American lineage are primarily isolated from swine and seals. Notably, all H3N2 subtype AIVs isolated from domestic poultry in China belong to the Eurasian lineage [9,10,11]. Important geographic regions associated with the epidemiological dissemination of H3 AIVs, including Alaska, Central Asia, and Chinese provinces, have been identified [2]. In China, H3 subtype AIVs are extensively distributed in poultry, with waterfowl serving as natural reservoirs [12,13]. Since 2000, surveillance data have revealed that combinations of H3 with N1-N8 subtypes are present in both poultry and wild birds, with H3N2 and H3N8 subtypes being predominant [9,12]. From 2014 to 2017, environmental samples collected from live poultry markets (LPMs) and poultry farms in China were analyzed, and the overall positive rate of H3 subtype AIVs was 0.3%, with H3N2 emerging as the dominant subtype [3]. Similarly, continued surveillance for AIVs in wild birds from 2003 to 2021 identified H3N2 AIVs as the predominant subtype of H3 AIVs [14]. Recent studies have reported a relatively high prevalence of the H3 subtype AIV in chickens in Shandong Province, with a positivity rate of 9.98% [15]. These findings indicate that H3N2 subtype AIVs are the predominant strain among H3 subtype AIVs in poultry populations.

Frequent gene reassortments and mutations in H3 subtype AIVs increase their replicative capacity and adaptability in mammals, thereby contributing to cross-species transmission. In recent years, novel reassorted H3 subtype AIVs have been demonstrated to increase pathogenicity in poultry and the ability to infect mammals [16,17]. In 2021, H3N8 reassortants from domestic chickens with respiratory symptoms were found to be genetically close to H3N8 strains that caused human infection events. These viruses may be derived from waterfowl-origin H3Nx AIVs, wild bird-origin HxN8 in North America, or chicken-origin H9N2 AIVs [16,18,19]. Nevertheless, the internal genes of duck-origin H3N2 AIVs are closely related to those of H5N8 viruses, which are capable of replicating in mouse lungs and nasal turbinates without prior adaptation [20]. Similarly, H3N3 subtype AIVs were generated from chickens in 2022 via gene reassortment involving H9N2, H3N8, and H10N3 AIVs, which are prevalent in domestic poultry. These novel reassortants demonstrated a wide range of tissue tropisms and exhibited efficient replication in the upper respiratory tract of SPF chickens [21]. These findings underscore the urgent need to evaluate the evolutionary trajectories and pathogenic potential of circulating H3 strains. Therefore, sustained surveillance of H3 subtype AIVs is essential to elucidate their adaptability and pathogenicity in mammals and poultry, which is critical for preventing avian influenza pandemics and decreasing spillover transmission to humans.

Guangxi, situated in southern China, functions as a significant center for avian trade activities and serves as a critical area for the breeding of migratory birds. Nonetheless, the genetic and pathogenicity characteristics of H3N2 AIVs in avian populations within Guangxi remain unclear. In this study, we analyzed the genetic evolution and molecular characteristics of nine duck-origin H3N2 AIVs obtained from LPMs in Guangxi, southern China. Furthermore, experimental infections were conducted in SPF chickens and BALB/c mice to assess the pathogenicity of these viruses.

## 2. Materials and Methods

### 2.1. Ethics Statements and Facilities

All work in this study was approved by the biosafety committee of Guangxi University (GXUKE2021-02; 16 March 2021). All animal experiments and experimental protocols were certified by the Animal Ethics Committee of Guangxi University (Guangxi, China), and the Animal Experimental Ethics Review Number was “GXU-2025-197”.

### 2.2. Virus Isolation and Identification

**Figure 1:**
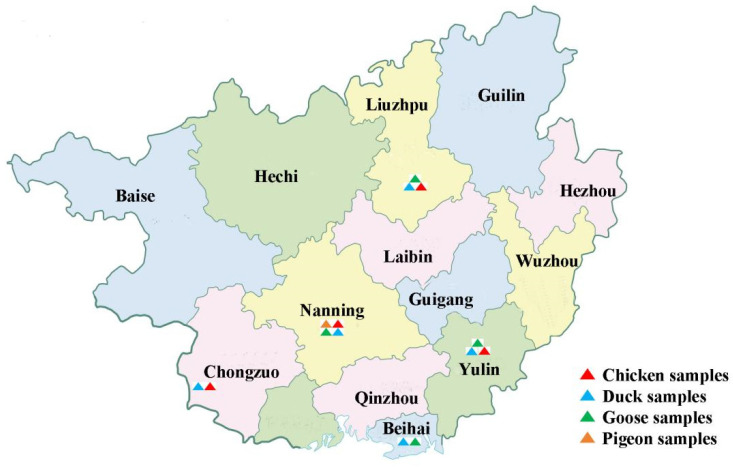
The sampling locations of poultry in Guangxi. The locations of poultry sampling in Guangxi were mapped using a standard map obtained from the Standard Map Service System (online website: https://bzdt-ch-mnr-gov-cn.vpn.gxu.edu.cn:8118/) (accessed on 28 August 2025), with subsequent processing performed in Adobe Photoshop CC 2018.

Between 2022 and 2024, oropharyngeal and cloacal swab samples were randomly obtained from healthy poultry in live bird markets in Guangxi, southern China (Figure 1). Swabs were preserved in phosphate-buffered saline (PBS) containing 1000 U/mL penicillin, 1000 U/mL streptomycin, and 20% glycerol. These samples were transported on ice and subsequently stored at −80 °C. Following the Chinese National Standard GB/T 18936-2020, all swab samples were centrifuged at 3000× *g* for 10 min at 4 °C [22]. For each sample, 0.2 mL of supernatant was inoculated into five 9-day-old embryonated specific-pathogen-free (SPF) chicken eggs via the allantoic cavity. The eggs were incubated at 37 °C and candled at 12 h intervals starting from 18 h post-inoculation (hpi). Allantoic fluid was harvested from embryos that died after 18 hpi and from those that remained alive at 72 hpi. Hemagglutination (HA) activity was tested using 1% chicken red blood cell suspension according to OIE standard procedures [23]. HA-positive samples were subsequently screened for H3N2 subtype AIV by RT-PCR. Positive isolates were propagated and purified by additional passage in 9-day-old SPF chicken eggs. A total of nine H3N2 AIVs isolated from ducks were obtained (Table 1).

To evaluate the 50% egg infective dose (EID_50_) of nine isolates, 10-fold serial dilutions of purified allantoic fluid were prepared using PBS, with the dilutions ranging from 10^−1^ to 10^−10^. A volume of 100 μL from each dilution was inoculated into five 9-day-old SPF chicken eggs, which were subsequently incubated at 37 °C for 72 h. Virus infection was tested by HA assay, and the EID_50_ value was calculated via the Reed-Muench method [24]. The EID_50_ values of the H3N2 isolates in this study are shown in Table 1. Specific pathogen-free (SPF) chickens and 9-day-old chicken embryos were purchased from Guangdong Xinxing Wens Foodstuff Group Co., Ltd. (Yunfu, China), while BALB/c mice were obtained from Nanning Yancheng Biotechnology Co., Ltd. (Nanning, China).

### 2.3. Genetic and Phylogenetic Analyses

The viral RNA was extracted using the RNAfast200 RNA Extraction kit (Fastagen, Shanghai, China) and subsequently converted into cDNA via reverse transcription. The eight gene segments of AIVs were amplified via RT-PCR using universal primers for influenza viruses, as described in a previous study [25]. All the PCR products were subjected to Sanger sequencing by Sangon Biotech Co., Ltd. (Shanghai, China). Nine strains isolated from ducks were identified as H3N2 subtype AIVs. The raw sequencing reads were quality-trimmed and assembled into contigs using the SeqMan tool from Lasergene 7.1 (DNASTAR 7.1, Madison, WI, USA). The assembled contigs were then manually inspected and edited to resolve ambiguities and generate consensus sequences for each viral genome. The complete viral genome nucleotide sequences were subsequently searched for homologous sequences via the BLAST tool (BLAST 2.17.0) provided by the National Center of Biotechnology Information (NCBI). All nucleotide sequences of the nine H3N2 AIVs isolated from ducks have been submitted to the GenBank database with accession numbers ranging from PV960403 to PV960474 (Supplementary Table S1). The nucleotide sequences of the reference strains were obtained from the NCBI and GISAID databases for genetic and phylogenetic analyses (Supplementary Table S1). The construction of phylogenetic trees was conducted with MEGA-X via the maximum likelihood method with the general time-reversible model, incorporating a bootstrap analysis of 1000 replicates [26]. The genetic distance was represented by the horizontal distances in the phylogenetic trees. The phylogenetic tree was annotated and visualized via the online tool EvolView, which is available at https://evolgenius.info//evolview-v2 (accessed on 1 July 2025). The molecular characteristics of the nine strains were compared with those of the reference strains A/Aichi/2/1968 (H3N2) and A/Goose/Guangdong/1/1996 (H5N1).

### 2.4. Pathogenicity in Chickens

To evaluate the pathogenicity of novel H3N2 AIVs in chickens, three H3N2 strains (LZD44, NND98, and NND100) were selected for infection studies. The experimental design has been previously described [27,28]. Briefly, sixty five-week-old SPF chickens were randomly assigned to four groups, including the control group, the NND98-inoculated group, the NND100-inoculated group, and the LZD44-inoculated group, with each group comprising 15 chickens. Each chicken in the inoculated groups was inoculated intranasally with 10^7^ EID_50_/0.2 mL H3N2 viruses (NND98, NND100, or LZD44). Concurrently, each chicken in the control group was challenged with 0.2 mL of PBS via the same route. To investigate the transmission of these isolates, three naive chickens, referred to as the contacted chickens, were inoculated intranasally with 0.2 mL of PBS and introduced to each group of infected chickens at 24 h post-inoculation (hpi). All chickens were monitored for clinical symptoms until 14 day post-inoculation (dpi). At 3 and 5 dpi, three chickens per inoculated group were euthanized to determine viral titers across multiple tissues. The euthanasia procedures for the SPF chickens were conducted in accordance with the American Veterinary Medical Association (AVMA) Guidelines for the Euthanasia of Animals (2020) [29]. In brief, the SPF chickens’ legs were grasped, and their neck were stretched by pulling on the head and applying a rotational force to the skull from the ventral to the dorsal side. The chickens were subsequently sterilized by immersion in 75% alcohol before being dissected. Tissues, including the brain, spleen, kidney, lung, liver, intestine, trachea, and pancreas, were collected and then stored at −80 °C. The same treatment was conducted on dead chickens in the control and contact groups. Viral shedding in chickens was monitored through the collection of oropharyngeal and cloacal swabs at 1, 3, 5, 7, 9, 11, and 14 dpi. Tissue homogenates for viral titration were prepared by homogenizing 1 g of tissue in 1 mL of PBS supplemented with 1000 U/mL penicillin-streptomycin. The supernatant was obtained by centrifugation at 5000× *g* for 10 min. Subsequently, the harvested supernatants were diluted in a series of 10-fold steps ranging from 10^0^ to 10^−8^. Each dilution, with a volume of 100 µL, was injected into 9–11-day-old SPF embryonated eggs, which were then incubated at 37 °C for 48 h. The Reed-Muench method was used to determine viral titers expressed as log_10_EID_50_/0.1 mL [30]. Additionally, all surviving chickens at 14 dpi were euthanized for serum collection, and seroconversion was confirmed via the hemagglutination inhibition (HI) test. HI antibody titers in the inoculated group of chickens were determined using 4 HAU of the homologous antigens NNC98, NNC100 and LZD44. Serum samples were serially 2-fold diluted, mixed with 4 HAU of viral antigen and incubated at room temperature for 30 min. Then 1% chicken red blood cells was gently mixed with the antigen–antibody complex and incubated at room temperature for 40 min. HI antibody titer ≤ 1:8 were considered negative and HI antibody titer ≥1:16 were considered positive [22].

### 2.5. Pathogenicity in Mice

In this study, sixty female SPF BALB/c mice (5 weeks old) were randomly allocated to four experimental groups, including the control group and three inoculated groups, with 15 individuals per group. In each group, the mice were anesthetized via isoflurane and intranasally inoculated with 10^6^ EID_50_/50 μL of H3N2 virus (NND98, NND100, or LZD44). Concurrently, the mice in the control group were inoculated with 50 μL of PBS via the same method. At 3 and 5 dpi, three mice (per inoculated group) were euthanized for viral titer assessment across tissues, including the brain, spleen, kidney, lung, and nasal turbinates. Viral titers in all tissues were measured as described above. Daily monitoring for weight loss and clinical symptoms was conducted on the mice in all four groups until 14 dpi. The serum of the remaining mice was collected at 14 dpi to assess seroconversion. Briefly, serum samples were pre-treated with a receptor-destroying enzyme (RDE) () as follows: 50 μL of serum was mixed with 200 μL of RDE and incubated at 37 °C for 18 h. Subsequently, 250 μL of 1.5% sodium citrate solution was added, and the mixture was incubated at 56 °C for 30 min to inactivate residual RDE activity. To remove nonspecific agglutinins, 50 μL of 50% chicken red blood cells was added to 500 μL of RDE-treated serum, and the mixture was incubated at 4 °C for 1 h with intermittent shaking. Following centrifuging at 1000× *g* for 10 min, the supernatant was collected for the HI test. HI antibody titers ≥ 1:20 were regarded as positive [22].

### 2.6. Histopathological Examination

To assess pulmonary histopathological changes, three chickens or mice were randomly selected from each experimental group (NND98-inoculated, NND100-inoculated, LZD44-inoculated, and control groups) and euthanized at 3 dpi. Lung tissues were harvested, fixed in 10% formalin, embedded in paraffin, sectioned, and subsequently stained with hematoxylin and eosin (H&E). Histopathological examination was performed using a light microscope at a magnification of ×100. Lesions in the lung tissues were evaluated using a semi-quantitative scoring system based on predefined pathological criteria, including the integrity of alveolar/parabronchial structures, epithelial cell degeneration/necrosis, capillary congestion, and inflammatory cell infiltration. Each parameter was rated on a scale from 0 to 4 (0 normal; 1, minimal; 2, moderate; 3, marked; 4, severe) [31]. For each specimen, three to five randomly selected microscopic fields were analyzed. Two independent, blinded investigators performed the scoring, and the mean score was used for statistical analysis. Quantitative morphometric analysis was conducted using ImageJ/Fiji software (Fiji 1.54) [32]. For each group, lung pathological sections obtained from three animals were analyzed, with three distinct fields of view evaluated per section.

### 2.7. Statistical Analysis

Data were expressed as means ± SD, and two-way ANOVA was used for multiple comparisons via GraphPad Prism 8.0 (GraphPad Software Inc., San Diego, CA, USA) with one independent variable. Statistical significance is represented by * *p* < 0.05; ** *p* < 0.01; *** *p* < 0.001; and **** *p* < 0.0001.

## 3. Results

### 3.1. Genetic Analysis of H3N2 Subtype AIVs

**Figure 2:**
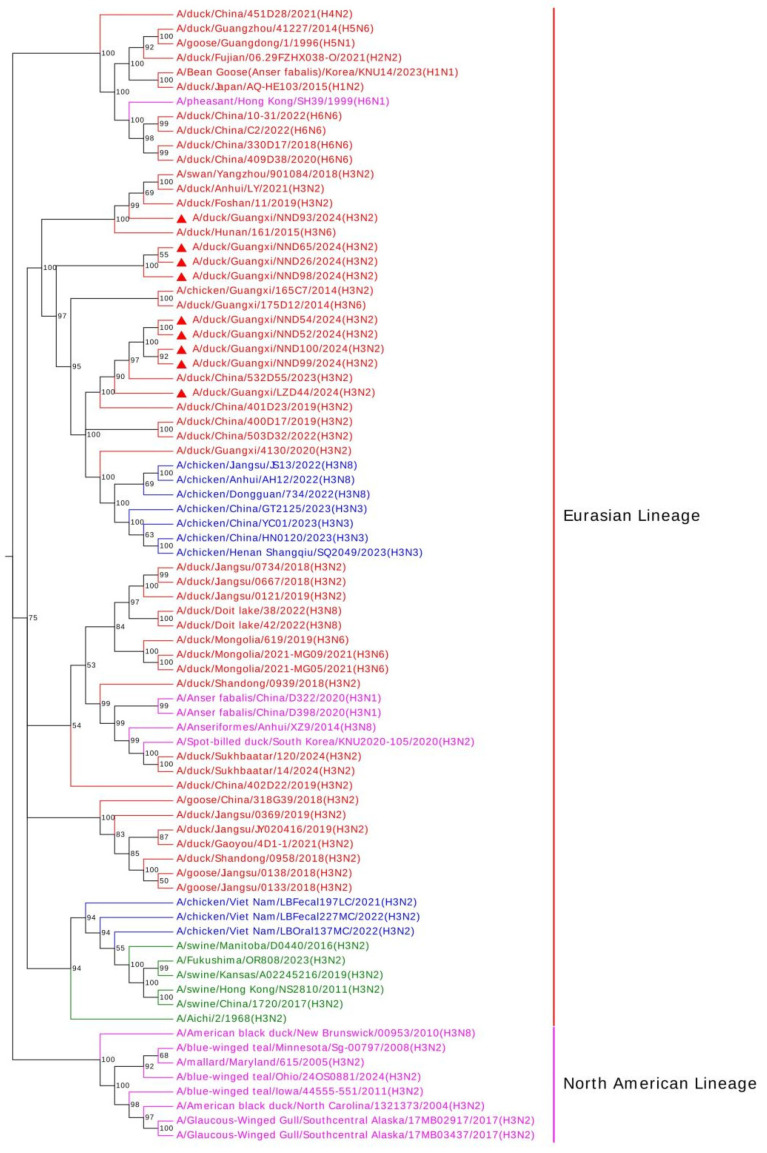
Phylogenetic tree of the *HA* genes of the nine isolates. Isolates in this study are marked with red triangles. The nucleotide sequences from chickens, waterfowl, wild birds, mammals, and others are marked with blue, red, magenta, green, and black, respectively. Phylogenetic trees were constructed with MEGA-X via the maximum likelihood method with bootstrap analysis (1000 replicates). Horizontal distances in phylogenetic trees are proportional to genetic distance.

Between 2022 and 2024, a total of 386 samples were collected from healthy poultry in LPMs in Guangxi. The overall positivity rate for AIV was 11.92%, yielding 46 isolates. These isolates comprised viruses of the H3, H6, and H9 subtypes. Notably, nine H3N2 AIV strains were isolated from ducks. Phylogenetic analysis of the *HA* gene indicated that all nine isolates clustered within the Eurasian lineage, were closely related to H3 subtype viruses from Chinese poultry, and were distinct from those originating from humans and swine (Figure 2). The *HA* genes of these H3N2 viruses presented nucleotide similarities ranging from 90.4% to 100% (Table 2). BLAST analysis revealed that the *HA* genes of strains NND26, NND65, and NND98 presented the highest similarity to those of A/chicken/Guangxi/165C7/2014 (H3N2), whereas those of strains LZD44, NND52, NND54, NND99, and NND100 were most similar to those of A/duck/China/532D55/2023 (H3N2). The *HA* gene of strain NND93 had the highest similarity with that of A/duck/Foshan/11/2019(H3N2) (Supplementary Table S2).

**Figure 3:**
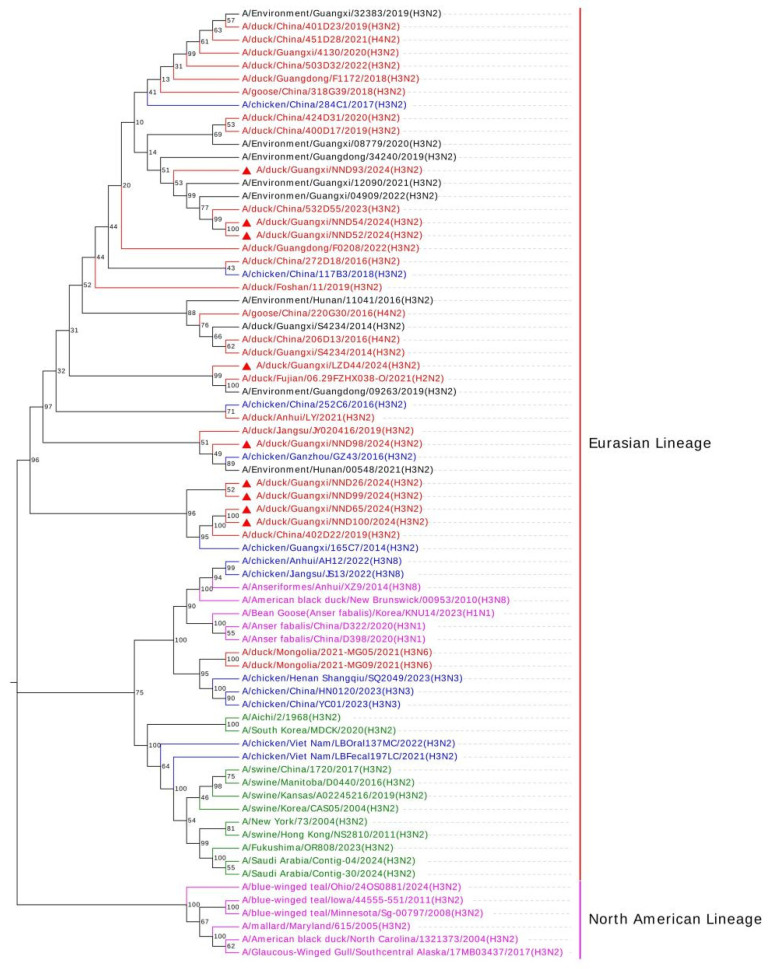
Phylogenetic tree of the *NA* genes of the nine isolates. Isolates in the study are marked with red triangles. The nucleotide sequences from chickens, waterfowl, wild birds, mammals, and others are marked with blue, red, magenta, green, and black, respectively.

All *NA* genes of the nine H3N2 AIVs belong to the Eurasian lineage (Figure 3), with nucleotide sequence similarities ranging from 89.8% to 100% among them (Table 2). Notably, the *NA* gene of strain LZD44 was closely related to that of an H2N2 strain (A/duck/Fujian/06.29FZHX038-O/2021), with a nucleotide similarity of 95.5% (Supplementary Table S2). In contrast, the remaining isolates clustered with H3N2 strains circulating in the environment and poultry in China, with nucleotide similarities ranging from 94.8% to 98.4% (Supplementary Table S2).

**Figure 4:**
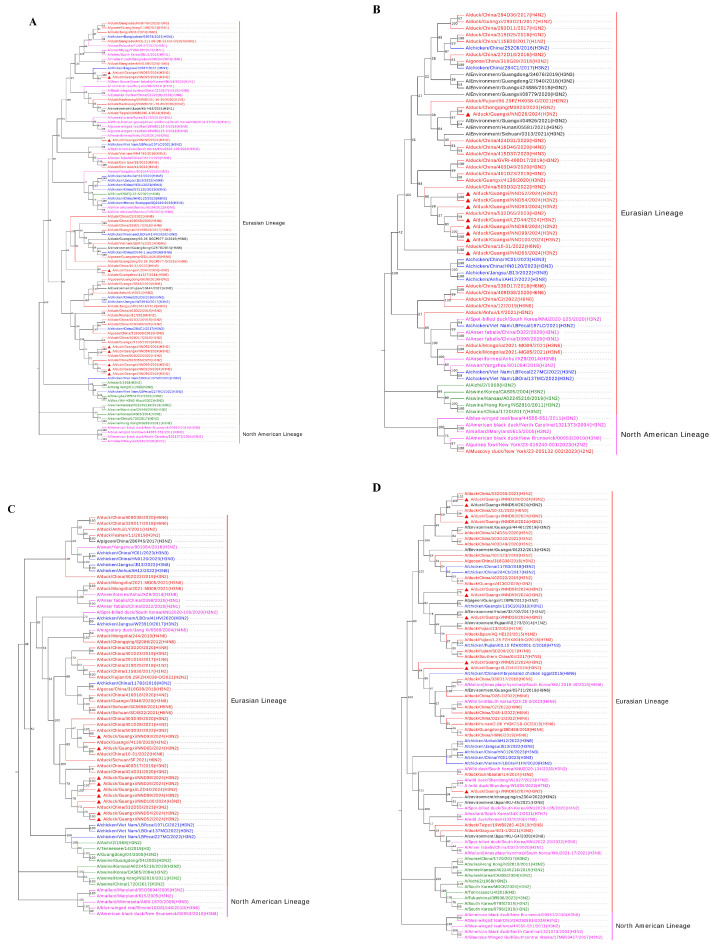

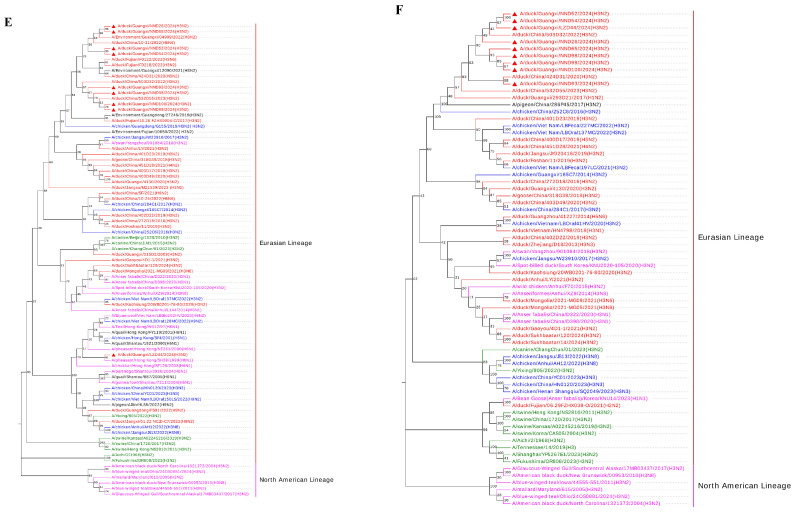
Phylogenetic tree of the internal genes of the nine isolates. (**A**) *PB2*, (**B**) *PB1*, (**C**) *PA*, (**D**) *NP*, (**E**) *M*, (**F**) *NS*. Isolates in the study are marked with red triangles. The nucleotide sequences from chickens, waterfowl, wild birds, mammals, and others are marked with blue, red, magenta, green, and black, respectively.

The six internal genes, *PB2*, *PB1*, *PA*, *NP*, *M*, and *NS*, of the nine viruses were all clustered into the Eurasian lineage (Figure 4A–F). These internal genes displayed high homology, with nucleotide similarities for the *PB2*, *PB1*, *PA*, *NP*, *M*, and *NS* genes ranging from 97.2% to 100%, 98.2% to 100%, 99.2% to 99.9%, 96.0% to 99.6%, 97.2% to 100%, and 96.6% to 100%, respectively (Table 2). Notably, the *PB2* gene of the LZD44 strain was closely related to that of the highly pathogenic H5N6 virus that emerged in Vietnam and southern China (Figure 4A). Similarly, *PB2* of the NND65 and NND26 strains were grouped with H5N1 viruses from poultry and wild birds in Japan, Korea, China, and Bangladesh, with nucleotide similarities of 92.0% to 99.1%. *PB2* of the remaining strains was closely related to H3 subtype AIVs from Chinese poultry, with nucleotide similarities ranging from 93.5% to 99.3% (Figure 4A). The *PB1* gene of the NND26 strain was clustered with a group comprising H2, H3, and H4 AIVs from ducks and environmental samples in China (Figure 4B), which presented the highest similarity to the H2N2 strain (A/duck/Fujian/06.29FZHX038-O/2021(H2N2)) (Supplementary Table S2). *PB1* of the NND65 strain presented a high nucleotide similarity of 98.3% with that of an H6N6 strain (A/duck/China/10-31/2022(H6N6)) (Supplementary Table S2). Moreover, the *PB1* genes of the other isolates demonstrated close genetic affinity to a duck-origin H3N2 strain (A/duck/China/532D55/2023(H3N2)), with nucleotide similarities ranging from 98.3% to 99.6% (Supplementary Table S2). The *PA* genes of the NND93 and NND65 strains were closely related to those of the H3N2 strains (A/duck/China/503D32/2022(H3N2) and A/duck/Guangxi/4130/2020(H3N2)), respectively (Figure 4C). The *PA* genes of the remaining strains were closely related to those of the other strains (A/duck/China/400D17/2019(H3N2)), with nucleotide similarity ranging from 98.6% to 99.1% (Supplementary Table S2). With respect to the *NP* gene, the NND65 strain was closely related to H3 and H7 AIVs from wild birds, predominantly in Japan, Korea, and China (Figure 4D), suggesting that wild bird migration may facilitate gene reassortment between the H3 and H7 AIV subtypes. The *NP* genes of the LZD44 and NND52 strains were closely related to those of H6N6 strains from wild birds in Korea and poultry in China, with nucleotide similarities ranging from 92.1% to 95.7% (Figure 4D). *NP* genes of the NND26, NND98 and NND99 strains were grouped with H1, H3, and H7 AIVs circulating in Japan and southern China (Figure 4D). The *NP* genes of these viruses displayed nucleotide similarities ranging from 94.8% to 97.6%. In contrast, the *NP* genes of the remaining strains (NND54, NND93, and NND100) were closely related to those of (A/duck/China/532D55/2023(H3N2)), with nucleotide similarities ranging from 96.1% to 99.5% (Supplementary Table S2). The *M* gene of LZD44 fell into a group containing H3, H6, and H9 viruses from Vietnam and China (Figure 4E), showing the highest nucleotide similarity of 100% with A/pheasant/Hong Kong/SH39/99 (H6N1) (Supplementary Table S2). The *M* genes of the remaining strains were closely related to H3N2 AIVs from ducks and environmental samples in China (Figure 4E), with nucleotide similarities ranging from 98.3% to 99.4%. The *NS* genes of all nine strains were closely related to those of the H3N2 strain (A/duck/China/503D32/2022(H3N2)) (Figure 4F), which shares nucleotide similarity from 98.46% to 99.29% (Supplementary Table S2).

In summary, phylogenetic analysis revealed H3N2 AIVs circulating in southern China are characterized by complex gene constellations resulting from extensive reassortment.

### 3.2. Molecular Characterization of H3N2 AIVs

To investigate the molecular characterization of the nine strains, amino acid sequence alignment was conducted between these viruses and the reference strain A/Aichi/2/1968(H3N2). The HA protein of all the strains presented ^340^PEKQTR↓GLF^348^ at the cleavage site, which aligns with the characterization of LPAIVs (Table 3). Six potential N-linked glycosylation sites were identified in the HA protein of the nine strains at positions ^22^NDS^24^, ^38^NGT^40^, ^54^NAT^56^, ^181^NVT^183^, ^301^NGS^303^, and ^499^NGT^501^ (Table 3). All nine viruses displayed the QSG motif at residues 226–228 (H3 numbering) within the receptor-binding site, a characteristic indicative of a preference for binding to the avian-type receptor (α-2,3-linked sialic acid) [33]. Notably, although this avian-type specificity, these viruses also carried the I155T mutation in HA (Table 4), a substitution previously associated with enhanced affinity for human-type receptors (α-2,6-linked sialic acid) [34].

For the NA protein, seven potential glycosylation sites were conserved across the isolates, comprising the motifs NIT, NNT, NWS, NGT, NAT, NGT, and NWS (Table 5). However, no deletions were observed in the NA stalk region (positions 63–65), a feature often linked to enhancing mammalian adaptation [35]. Furthermore, none of the isolates possessed well-characterized neuraminidase inhibitors resistance mutations, such as E119D, H274Y, and R292K (Table 4) [36,37]. Interestingly, a V263I mutation under positive selection was identified in the NA protein [38,39]; four of the nine isolates contained I at this position, while the remaining five harbored V.

Amino acid substitutions within the internal genes of AIVs that modulate pathogenicity and adaptation to mammalian hosts have been identified [36]. Although the majority of sites retained avian virus signatures, all nine H3N2 AIVs possessed several amino acid mutations previously associated with increased polymerase activity and virulence in mice. These mutations include PB2-K389R, PB2-V598T, PB1-E198K, PB1-D622G, PA-S37A, PA-N383D, NS1-P42S, M1-N30D, M1-T215A, and NS1-D92E (Table 4) [36,40].

In the PB2 gene, all nine H3N2 AIVs maintained avian virus signatures at several critical sites, such as 256D, 271T, 292I, 588A, 627E, and 701D (Table 4). Notably, the LZD44 strain uniquely harbored the PB2-MVV motif, comprising three substitutions (I66M, I109V, and I133V), which has been regarded as a prerequisite for the acquisition of additional mammalian-adaptive mutations [41,42]. This strain also carried PB2-V255I, a substitution associated with increased pathogenicity [43,44]. Moreover, the mutations of PB2-V338I were only present in the LZD44 strain, which was previously identified in H5N8 AIVs and H3N2 canine influenza viruses (Table 4) [45,46].

In the NP gene, all isolates carried the NP-A184K mutation, a substitution linked to increased virulence of H5N1 viruses in chickens (Table 4) [47]. Interestingly, five of the nine strains contained the NP-V105M mutation, which has been associated with increased virulence of the H9N2 virus in mice [48,49]. Furthermore, two of nine strains carried the NP-I353V mutation, which is implicated in enhanced virulence of H1 and H5 viruses in mice (Table 4) [50].

Consequently, while these H3N2 viruses predominantly exhibited avian signatures at most sites, some viruses harbored a few mammalian-adaptive mutations, suggesting a potential for enhanced pathogenicity in mammalian hosts.

### 3.3. Pathogenicity and Transmission of the H3N2 AIVs in SPF Chickens

In the present study, phylogenetic analysis revealed that the eight gene segments of the nine strains exhibited complex reassortment origins. Specifically, the LZD44 strain derived its gene segments from multiple AIV subtypes, including H3, H5, and H6. The gene segments of the NND98 strain originated from H1 and H3 subtypes, whereas those of the NND100 strain were exclusively derived from the H3 subtype. Notably, these three strains possessed distinct combinations of mutations previously associated with AIV pathogenicity and mammalian adaptation. These mutations include PB2-MVV, PB2-V255I, PB2-I495V, NP-V105M, and NP-I352V (Table 4). Given that such genetic characteristics may influence the virulence of these viruses, the three representative strains (LZD44, NND98, and NND100) were selected for further pathogenicity testing.

The result showed that no obvious clinical symptoms or mortality were observed in the chickens throughout the experimental period. At 3 dpi, the LZD44 strain was detected in various tissues, including the brain, spleen, kidney, lung, trachea, and pancreas, with mean viral titers of 3.25–7 log_10_EID_50_/g (Table 6). However, the NND98 and NND100 strains exhibited restricted tissue tropism and were detected exclusively in respiratory tract tissues (lung and trachea), with mean viral titers of 3.5–3.83 log_10_EID_50_/g and 2.83–2.5 log_10_EID_50_/g, respectively (Table 6). Statistical analysis revealed that the replication of the LZD44 strain in the brain (*p* < 0.0001), spleen (*p* = 0.0031), lung (*p* < 0.05), and trachea (*p* < 0.05) was significantly greater than that of the NND98 and NND100 strains. At 5 dpi, the LZD44 strain achieved mean viral titers of 1.67–5.42 log_10_EID_50_/g in various tissues, including the brain, spleen, kidney, lung, intestine, trachea, and pancreas. The NND98 strain replicated to a mean titer of 2–4.42 log_10_EID_50_/g in tissues, including kidney, lung, liver, intestine, trachea, and pancreas. The NND100 strain primarily replicated in the lung and trachea, with mean titers ranging from 2.5 to 2.83 log_10_EID_50_/g (Table 6). The viral titers in the pancreas (*p* < 0.01) from the LZD44-inoculated group were significantly higher than those of the NNC98- and NNC100-inoculated group.

**Figure 5:**
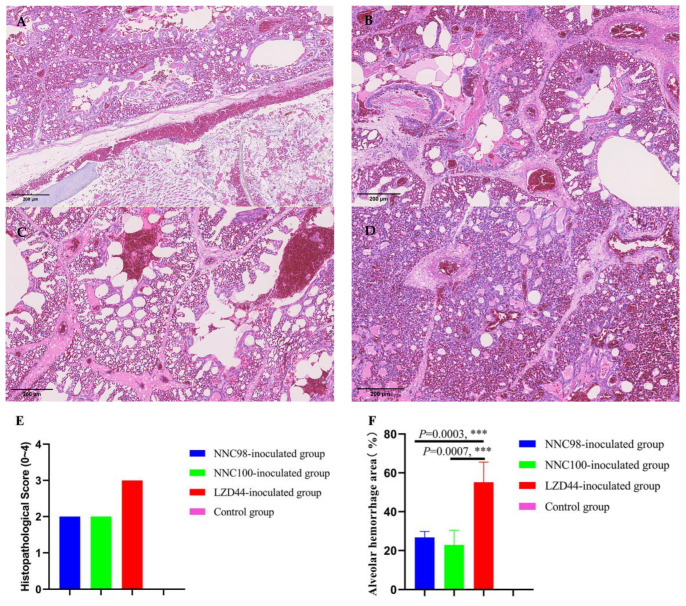
Histopathological lesions in the lungs of H3N2 AIV-infected SPF chickens. Lung sections from chickens in the NND98-inoculted (**A**), NND100-inoculated (**B**), LZD44-inoculted (**C**), and control groups (**D**) were stained with H&E. Scale bar, 200 μm (original magnification, ×100). (**E**) Semi-quantitative scoring criteria for lung lesions (score 0–4). A score of indicates normal lung tissue with no inflammatory infiltration and architecture preserved. A score of 1 denotes minimal changes, characterized by focal infiltration and mild congestion. A score of 2 represents moderate alterations, with multifocal infiltration and mild septal widening. A score of 3 is indicative of marked changes, including diffuse infiltration, epithelial hyperplasia, and perivascular edema. A score of 4 reflects severe damage in the lung tissues, such as extensive consolidation, bronchiolar epithelial necrosis, and massive hemorrhage. The colors blue, green, red, and magenta represented the NNC98-inoculated, NNC100-inoculated, LZD44-inoculated and control groups, respectively. (**F**) The alveolar hemorrhage area was quantified using Image J/Fiji software. For each group, lung pathological sections obtained from three mice were analyzed, with three different fields of view evaluated per section. Differences among groups were determined using a one-way ANOVA, and statistical significance was represented by: *** *p* < 0.001, with a *p* value < 0.05 indicated as statistically significant. The colors blue, green, red, and magenta represented the NNC98-inoculated, NNC100-inoculated, LZD44-inoculated and control groups, respectively.

Furthermore, histopathological examination of lung tissues from the virus-inoculated chickens was assessed at 3 dpi (Figure 5A–C). In lungs, NND98 infection caused moderate lesions characterized by congestion and hemorrhage in the lamina propria of the mucosa as well as multifocal infiltration (Figure 5A,E; score: 2/4). In the NND100-inoculated group, moderate lesions including congestion, perivascular edema, and multifocal infiltration were observed (Figure 5B,E; score: 2/4). The LZD44 strain caused marked pathology, with perivascular edema and marked accumulation of erythrocytes within the parabronchial lumina (Figure 5C,E; score: 3/4). Quantification of alveolar hemorrhage area revealed that a significantly larger hemorrhage area in the LZD44-inoculated group compared with the NNC98-inoculated group (*p* = 0.0003) and the NNC100-inoculated group (*p* = 0.0007) (Figure 5F). No appreciable pulmonary lesions were presented in the control group (Figure 5D–F). Consequently, these histopathological findings demonstrated that all three representative H3N2 isolates induced pulmonary lesions in SPF chickens, with the LZD44 strain causing more pronounced pathology.

Viral shedding was assessed through the collection of oropharyngeal and cloacal swabs from all inoculated chickens at 1, 3, 5, 7, 9, 11, and 14 dpi. In the LZD44-inoculated group, the virus was consistently detected in oropharyngeal swabs within a 14-day observation period, whereas it was detected in cloacal swabs from 3 to 11 dpi. NND98 virus was detected in both oropharyngeal swabs and cloacal swabs from 1 to 11 dpi. Similarly, viral shedding of the NND100 virus in chickens was noted in both oropharyngeal and cloacal swabs from 1 to 14 dpi (Table 7). These results suggested that the H3N2 AIVs were shed through both the respiratory and digestive systems, with prolonged viral shedding observed in inoculated chickens.

To determine the transmission of H3N2 AIVs among chickens, three naive chickens (named contact chickens) were housed with inoculated chickens at 24 hpi. No significant clinical symptoms were found in the contacted chickens from 1 to 14 dpi. Viral shedding of the LZD44 and NND98 viruses in contact chickens was detected in oropharyngeal swabs and cloacal swabs from 3 to 11 dpi (Table 7). In contrast, shedding of the NND100 virus in contact chickens was detected in oropharyngeal swabs and cloacal swabs between 5 and 11 dpi (Table 7). These findings indicated that three viruses were capable of being transmitted from inoculated chickens to contact chickens via direct contact, but the shedding of the LZD44 and NND98 viruses in contact chickens persisted for a longer duration than did the shedding of the NND100 virus.

### 3.4. Infection and Pathogenic Characteristics of H3N2 Isolates in Mice

The pathogenicity of H3N2 AIVs in BALB/c mice (6 weeks old) was assessed through intranasal challenge with 10^6^ EID_50_/50 µL of each virus. No mortality or overt clinical signs occurred from 1 to 14 dpi. At 3 dpi, the NND98 virus was detected in five tissues (brain, spleen, kidney, nasal turbinate, and lung), with mean titers of 2.08–5.50 log_10_EID_50_/g (Table 8). The NND100 virus replicated in the kidney, nasal turbinate, and lung, with mean titers of 1.67–5.50 log_10_EID_50_/g (Table 8). The LZD44 virus replicates in various tissues, including the brain, kidney, nasal turbinate, and lung, with mean titers ranging from 1.83 to 7.08 log_10_EID_50_/g (Table 8). At 5 dpi, all three viruses replicated in five tissues, with mean viral titers ranging from 1.67 to 6.67 log_10_EID_50_/g (Table 8). Specifically, the LZD44 virus presented significantly greater viral loads in the lung and nasal turbinate than the NND98 and NND100 viruses did (*p* < 0.05) (Table 8).

**Figure 6:**
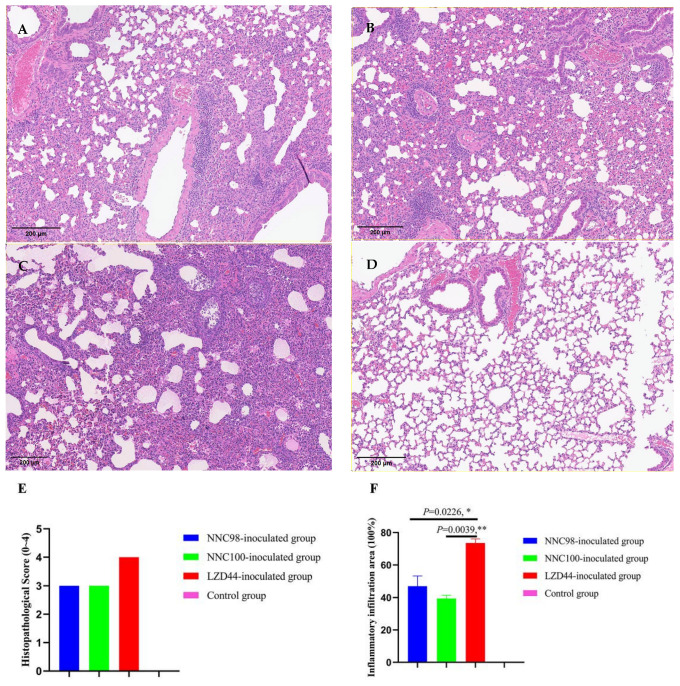
Histopathological lesions in the lungs of H3N2 AIV-infected mice. Lung sections from mice in the NND98-inoculted (**A**), NND100-inoculated (**B**), LZD44-inoculted (**C**), and control groups (**D**) were stained with H&E. Scale bar, 200 μm (original magnification, ×100). (**E**) Semi-quantitative scoring criteria for lung lesions (score 0–4). A score of 0 indicates normal lung tissue with no inflammatory infiltration and architecture preserved. A score of 1 denotes minimal changes, characterized by focal infiltration and mild congestion. A score of 2 represents moderate alterations, with multifocal infiltration and mild septal widening. A score of 3 is indicative of marked changes, including diffuse infiltration, epithelial hyperplasia, and perivascular edema. A score of 4 reflects severe damage in the lung tissues, such as extensive consolidation, bronchiolar epithelial necrosis, and massive hemorrhage. The colors blue, green, red, and magenta represented the NNC98-inoculated, NNC100-inoculated, LZD44-inoculated and control groups, respectively. (**F**) The inflammatory infiltration area was quantified using Image J/Fiji software. For each group, lung pathological sections obtained from three mice were analyzed, with three different fields of view evaluated per section. Differences among groups were determined using a one-way ANOVA, with a *p* value < 0.05 indicated as statistically significant, and statistical significance was represented by: * *p* < 0.05; ** *p* < 0.01. The colors blue, green, red, and magenta represented the NNC98-inoculated, NNC100-inoculated, LZD44-inoculated and control groups, respectively.

The histopathological analysis of lung tissues at 3 dpi revealed characteristic lesions across all inoculated groups. Extensive infiltration of inflammatory cells was observed within the bronchioles and surrounding alveolar spaces across the three inoculated groups (Figure 6A–C), with the LZD44 strain inducing the most pronounced inflammatory cell infiltration (Figure 6F). NNC98 infection resulted in marked lesions with diffuse infiltration and perivascular edema (Figure 6A,E; score: 3/4). Similarly, NNC100 induced marked lesions characterized by septal widening accompanied by diffuse infiltration of neutrophils. In Contrast, LZD44 caused severe alveolar damage, pronounced congestion, and numerous sloughed epithelial cell debris, serous exudate, and inflammatory cell infiltration within the bronchiolar lumina (Figure 6C,E; score: 4/4). No obvious lesions were detected in the control group (Figure 6D–F). These findings revealed that all three H3N2 isolates induced pulmonary damage in mice, with the LZD44 strain exhibiting higher virulence than the other strains.

**Figure 7:**
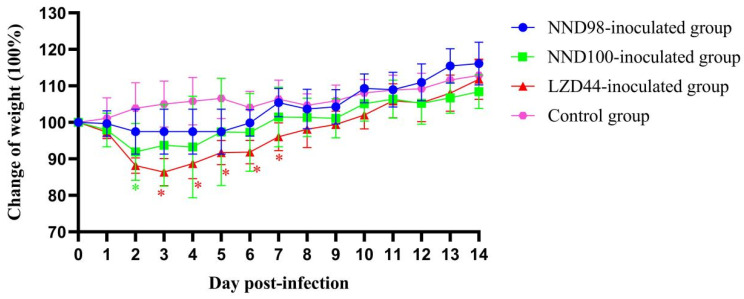
Weight changes in BALB/c mice infected with H3N2 AIVs. Body weights of mice in all four groups (n = 5 per group) were monitored daily until 14 dpi and expressed as percentages of the initial body weight. The difference between each virus-inoculated group and the control group was determined via two-way ANOVA using GraphPad Prism 8.0. Statistical significance is represented by * *p* < 0.05. The colors blue, green, red, and magenta represented the NNC98-inoculated, NNC100-inoculated, LZD44-inoculated and control groups, respectively.

Additionally, body weight monitoring revealed that the body weight of the mice challenged with the LZD44 virus decreased by 3.1–12% between 1 dpi and 7 dpi, which was significantly lower than that of the control group (*p* < 0.05). Conversely, the NND98-inoculated and NND100-inoculated groups presented slight body weight loss, with no significant difference compared with the control group (Figure 7). These results indicated that infection with the LZD44 virus induced more pronounced weight loss in mice than infection with the other viruses did, demonstrating its greater pathogenicity.

## 4. Discussion

H3 subtype AIVs, particularly H3N2 and H3N8 AIVs, are commonly detected in wild birds and poultry. On the basis of the HA gene, H3 subtype AIVs can be divided into Eurasian lineages and North American lineages [9,10]. All H3N2 AIVs isolated from China cluster within the Eurasian lineage [3,9]. H3N2 AIVs, which are frequently isolated from waterfowl, are widespread in poultry farms and LPMs in China [2,9,51,52]. Notably, H3N2 viruses originating from birds are also transmitted to dogs, leading to severe respiratory disease [8], highlighting their potential ability for cross-species transmission. In this study, nine H3N2 AIVs were isolated from ducks in LPMs in Guangxi. Phylogenetic analysis revealed that the eight gene segments from these nine H3N2 isolates clustered within the Eurasian lineage, exhibiting a close relationship with viruses from avian species and distinct from those from humans and swine. Additionally, certain internal genes, including PB2, M, and NP, were closely related to those of viruses from wild birds, indicating that wild bird migration may facilitate gene reassortment in H3N2 AIVs.

The evolutionary dynamics of H3N2 AIVs are notably complex and characterized by genetic diversity via gene mutations and segment reassortment with various AIV subtypes [3,27,53,54]. Consistent with the previous reports, the HA and NA genes of the nine H3N2 AIVs were closely related to those of H3 AIVs from poultry in China [55,56]. In contrast, the internal genes of these isolates displayed considerable diversity. Notably, the PB2 genes of the LZD44, NND65, and NND26 strains presented the highest sequence homology with H5 HPAIVs from ducks and wild birds across different geographical regions, including Korea, Japan, China, and Bangladesh. This finding revealed that frequent genetic exchange between H5 HPAIVs and waterfowl-origin H3N2 AIVs in these areas. However, such reassortment events are not limited to the PB2 genes. Several studies have revealed that the internal genes of some H3N2 AIVs are closely related to those of H5 HPAIVs, further indicating the occurrence of reassortment events between these viruses [13,20,57]. Furthermore, H5 subtype AIVs frequently acquire internal genes from LPAIVs, including those of the H3 subtype [58,59]. For example, H5N6 AIVs carrying a PB2 gene of H6, a PB1 gene of H3, and other internal genes derived from H5N1 have been circulating in China since 2016 [60]. Consequently, these data suggest that H3N2 subtype AIVs may serve as donors of internal genes for reassortment with highly pathogenic H5 subtype AIVs, potentially contributing to the emergence of novel viruses with altered pathogenicity and host range. Additionally, previous studies have shown that the internal genes of H3N2 AIVs are derived from various low-pathogenicity AIVs, including the H1, H2, H3, H4, H6, H7, H9, and H11 subtypes, underscoring the genetic complexity of these viruses [9,20,53,54]. In alignment with these findings, the internal genes of the nine H3N2 viruses were closely related to multiple LPAIVs, such as H1, H2, H3, H4, H6, H7, and H9 AIVs, suggesting frequent and intricate genetic reassortment events involving H3N2 AIVs in Guangxi. Such genetic exchange between H3N2 and other circulating AIVs contributes to the dynamic evolution of these viruses, potentially leading to pandemic emergence. Although H3N2 AIVs have not been associated with widespread human infections, their role as gene donors in the generation of reassortants with zoonotic potential warrants attention. Notably, the human infections with H3N8 AIVs, a novel reassortant generated via complex genetic exchange involving H3N2, wild bird H3N8, and poultry H9N2 viruses [16,61], highlight the ability of H3N2 viruses to contribute internal genes to emerging viruses that cross the species barrier. These findings underscore the need for continuous surveillance to monitor the spillover potential of emerging avian influenza viruses.

Molecular characterization of the nine H3N2 AIVs revealed critical features linked to host adaptation and pathogenicity. The HA cleavage site (^340^PEKQTR↓GLF^348^) in all nine isolates contained a single arginine residue, which is consistent with the characterization of LPAIVs [62]. Furthermore, the QSG motif at positions 226–228 in the receptor-binding site was detected in the nine viruses, indicating that they favor binding to avian-type receptors [33]. However, the I155T mutation in the HA protein, which is found in all nine H3N2 AIVs, potentially increases virulence in mice and promotes binding to human-type receptors [34]. In this study, the nine H3N2 viruses predominantly retained avian signatures at most sites, whereas several isolates harbored a few mammalian-adaptive mutations. PB2 protein, a key subunit of the viral polymerase complex, determines viral replication efficiency in various hosts [63]. Although the mammalian-adaptive residues PB2-627K and PB2-701N were absent in all isolates in this study, previous studies have demonstrated that these mutations can emerge following viral replication in mammalian hosts [33,34,64]. For instance, Li et al. [54] found that the PB2 E627K mutation in viruses recovered from H3N2 AIV-infected mice. Yu et al. [65] demonstrated that the presence of PB2-701N is in a mouse-adapted H3N2 avian virus. Acquisition of either of these residues in H3N2 viruses may be associated with a switch in human-type receptor specificity, highlighting their potential threat to humans. Additionally, other mutations in PB2 also increase viral polymerase activity in mammalian cell lines across multiple AIV subtypes. The PB2-MVV substitutions have been identified as an initial step for AIV to acquire mammalian pathogenicity, enabling the subsequent acquisition of lethal mammalian-adaptive mutations such as I147T, K339T, A588T, Q591R/K, E627K, or D701N [41,42]. Song et al. [66] demonstrated that introduction of the PB2-MVV modifications into an H9N2 vaccine strain enabled efficient replication in embryonated eggs without repeated passaging, thereby overcoming the limitations of conventional vaccine strains that may compromise immunogenicity. Notably, the PB2-MVV motif was uniquely identified in the LZD44 strain, suggesting its potential as a vaccine candidate. Another substitution, PB2-V255I, has been associated with increased pathogenicity of H1N1 AIVs in mice [43,44]. However, previous studies have shown that the introduction of this mutation into an H1N1 swine influenza virus did not significantly alter pathogenicity in mice [67]. Whether the PB2-V255I mutation contributes to the virulence of the LZD44 strain needs further investigation. In the NP gene, mutations associated with enhanced virulence in mammalian systems were also observed. The NP-V105M mutation, previously associated with increased virulence of H9N2 and H6N5 viruses in the mammalian system [48,49], was found in five of the nine viruses. Sequence analysis indicated that approximately one-seventh of the avian isolates carry NP-105M, whereas this residue is rarely found in human or swine isolates [49]. Furthermore, the LZD44 specifically harbored the NP-I353V substitution, a residue previously presented in the mouse-adapted viruses [50]. Thus, the exclusive presence of mammalian-adaptive mutations in the LZD44 strain suggests a distinct pathogenicity compared with the other H3N2 isolates in this study.

Research has suggested that H3N2 AIVs have restricted replication in chickens [13,20,62]. In China, an H3N2 AIV isolated from ducks has been shown to restrict replication in the intestine and kidney [13]. Similarly, chickens inoculated with H3N2 AIVs obtained from ducks in LPMs from Korea presented no obvious signs and limited viral replication in the upper respiratory tract [68]. In this study, no obvious clinical symptoms or mortality were observed in chickens throughout the experimental period. The LZD44 strain replicates in multiple tissues, including the brain, spleen, kidney, lung, intestine, trachea, and pancreas. In contrast, the NND98 strain was detected in the kidney, lung, liver, intestine, trachea, and pancreas, whereas the NND100 strain primarily replicated in the lung and trachea. All inoculated SPF chickens shed virus via respiratory and fecal routes, enabling efficient horizontal transmission to cohoused contacts. Furthermore, at 3 dpi, the LZD44 strain exhibited significantly greater replication in respiratory tissues than did the NND98 (*p* < 0.001) and NND100 strains (*p* < 0.05). These results indicate that the LZD44 strain has greater virulence in chickens than do the NND98 and NND100 strains. The source of the internal genes of the three viruses may explain the varying pathogenicity of these viruses in chickens. In particular, the PB2 gene of the LZD44 strain showed high homology with those of H5N6 HPAIVs. Cui et al. [68] reported that the internal genes of three H3N2 AIVs are closely related to those of H5N8 HPAIVs and that these viruses can replicate in multiple tissues of chickens, including the trachea, lung, pancreas, and intestine. These results were similar to those of our study. In 2023, the internal gene of the H3N6 subtype AIV, related to outbreaks in laying ducks, shared high homology with those of currently circulating H5 HPAIVs. This strain induced respiratory and neurological symptoms in SPF ducks, with a mortality rate reaching 40% [69]. Therefore, gene reassortment between H3N2 AIVs and HPAIVs may increase the pathogenicity of novel viruses in chickens, highlighting the necessity for continued monitoring of AIVs.

In BALB/c mice, three strains replicate in respiratory tissues without causing clinical signs. Three viruses replicate in multiple tissues, including the brain, spleen, kidney, nasal turbinates, and lung. In the LZD44-inoculated group, significantly greater viral loads were detected in respiratory tissues (nasal turbinates and lungs) at 5 dpi than in the NND98- and NND100-inoculated groups. Furthermore, significant body weight loss was observed in the LZD44-inoculated mice, whereas no such loss was detected in the NND98-inoculated and NND100-inoculated groups. The previously mentioned H3N2 AIVs can replicate in the nasal turbinates and lungs of inoculated mice without prior adaptation [20]. Additionally, an H3N2 AIV isolated from ducks in China was able to replicate in the lungs of mice and caused slight body weight loss. Genetic analysis revealed that its seven gene segments presented the highest sequence similarities with those of the H7 subtype influenza virus [13]. Li et al. found that twenty-one H3N2 AIVs could replicate in the upper respiratory tissues (nasal turbinates and lungs) of inoculated mice, with only seven of these viruses causing body weight loss. Thus, H3N2 AIVs mainly replicate in respiratory tissues in inoculated mice, and body weight loss is common in viruses that have recombined with HPAIVs, which was in accordance with our study. Furthermore, the PB2 protein is an important component of the IAV polymerase complex, and its amino acid changes are associated with virus replication and pathogenicity [63,70,71]. The three permissive substitutions of PB2-MVV acted as a prerequisite for the acquisition of additional mammalian adaptive mutations, including I147T, K339T, A588T, Q591R/K, E627K, or D701N [41,42]. Although these mammalian adaptive mutations were not detected in the LZD44 strain, the virus was able to replicate effectively in mice without prior adaptation, highlighting its potential for cross-species transmission. Otherwise, other mutations specifically exhibited in the LZD44 strain, including PB2-V255I, PB2-V338I, PB2-R353K, and PB2-I495V, may also play important roles in its pathogenicity. The mechanisms by which these PB2 mutations influence the virulence and mammalian adaptation of H3N2 AIVs warrant further investigation.

Previous studies have demonstrated that duck-origin AIVs are effectively transmitted via contact in chickens [33,68,72,73,74]. In this study, viral shedding of three strains was observed in both inoculated and contacted chickens, which aligns with these findings. Multiple lineages of H3N2 influenza viruses are commonly detected in animals [8,33], and further studies have shown that H3N2 AIVs can bind to both human-type and avian-type receptors. Consequently, the receptor binding specificity of the nine strains needs further analysis.

Furthermore, H3N2 AIVs have been shown to be transmitted efficiently to guinea pigs and ferrets via respiratory droplets. Respiratory droplet transmission studies using guinea pigs are common, as influenza viruses typically cause no significant pathogenic damage in these animals. However, infected guinea pigs shed large quantities of the virus through direct contact and droplet transmission [33,75]. In contrast, the infection study involving the three strains was performed on BALB/c mice, which are smaller in size and exhibit lower levels of viral shedding, thereby making detection more challenging. Additionally, several mutations in the HA protein may increase the affinity of the virus for human-type receptors and contribute to its transmission in mammals [36,76]. A transmission study revealed that the G228S and Q226R mutations of HA are associated with increased transmission of H3N2 AIVs in guinea pigs via respiratory droplets, which are not present in the nine strains [33,72].

Our study focused on the genetic evolution and pathogenicity of H3N2 AIVs in Guangxi, providing a scientific foundation for developing control strategies to mitigate public health threats.

## 5. Conclusions

This study provides critical insights into the genetic evolution and pathogenicity of H3N2 subtype AIVs isolated from LPMs in southern China. All nine isolates exhibited complex reassortment events involving multiple AIV subtypes, indicating the intricate genetic diversity of H3N2 viruses circulating in Guangxi. Notably, three representative viruses could replicate in certain tissues of chickens and mice without prior adaptation, emphasizing the potential risk for cross-species transmission.

## Figures and Tables

**Table 1:** The H3N2 avian influenza viruses isolated from LPMs in southern China.

No.	Strain	Location	Host	EID_50_
1	A/duck/Guangxi/NND26/2024(H3N2)	Nanning	Duck	10^−5.5^
2	A/duck/Guangxi/LZD44/2024(H3N2)	Liuzhou	Duck	10^−8.0^
3	A/duck/Guangxi/NND52/2024(H3N2)	Nanning	Duck	10^−5.5^
4	A/duck/Guangxi/NND54/2024(H3N2)	Nanning	Duck	10^−5.167^
5	A/duck/Guangxi/NND65/2024(H3N2)	Nanning	Duck	10^−5.375^
6	A/duck/Guangxi/NND93/2024(H3N2)	Nanning	Duck	10^−5.5^
7	A/duck/Guangxi/NND98/2024(H3N2)	Nanning	Duck	10^−6.375^
8	A/duck/Guangxi/NND99/2024(H3N2)	Nanning	Duck	10^−5.375^
9	A/duck/Guangxi/NND100/2024(H3N2)	Nanning	Duck	10^−5.628^

**Table 2:** The nucleotide and amino acid sequence homology of the eight gene segments among the isolates.

Gene	Nucleotide Sequence		Amino Acid Sequence	
	ORF/bp	Similarity (%)	ORF/aa	Similarity (%)
*HA*	1701	90.4~100	566	96.1~100
*NA*	1410	89.8~100	469	95.1~100
*PB2*	2280	86.7~99.9	759	97.2~100
*PB1*	2274	96.0~99.8	757	98.2~100
*PA*	2151	96.8~99.9	716	99.2~99.9
*NP*	1495	88.0~98.7	498	96.0~99.6
*M*	982	93.0~99.8	252	97.2~100
*NS*	844	98.5~100	237	96.6~100

**Table 3:** The cleavage and glycosylation sites of the HA protein in this study.

Strain	Cleavage Sites ^①^	Glycosylation Sites ^①^					
	340–348	22–24	38–40	54–56	181–183	301–303	499–501
NND26	PEKQTR↓GLF	NDS	NGT	NAT	NVT	NGS	NGT
LZD44	PEKQTR↓GLF	NDS	NGT	NAT	NVT	NGS	NGT
NND52	PEKQTR↓GLF	NDS	NGT	NAT	NVT	NGS	NGT
NND54	PEKQTR↓GLF	NDS	NGT	NAT	NVT	NGS	NGT
NND65	PEKQTR↓GLF	NDS	NGT	NAT	NVT	NGS	NGT
NND93	PEKQTR↓GLF	NDS	NGT	NAT	NVT	NGS	NGT
NND98	PEKQTR↓GLF	NDS	NGT	NAT	NVT	NGS	NGT
NND99	PEKQTR↓GLF	NDS	NGT	NAT	NVT	NGS	NGT
NND100	PEKQTR↓GLF	NDS	NGT	NAT	NVT	NGS	NGT

^①^: Location H3 numbering relative to A/Aichi/2/1968 (H3N2). ↓: This arrow is represents the general notation for the cleavage site of the HA protein.

**Table 4:** The key amino acid sites of the H3N2 subtype AlVs in this study.

Protein	Phenotypic Effect	Substitution	NND26	LZD44	NND52	NND54	NND65	NND93	NND98	NND99	NND100
HA ^①^	Increased virus binding to α-2,6-linked sialic acid receptors	I155T	T	T	T	T	T	T	T	T	T
		N193S	N	N	N	N	N	N	N	N	N
		Q226L	Q	Q	Q	Q	Q	Q	Q	Q	Q
		S227N	S	S	S	S	S	S	S	S	S
		G228S	G	G	G	G	G	G	G	G	G
NA ^①^	Reduced susceptibility to neuraminidase inhibitors	E119D	E	E	E	E	E	E	E	E	E
		H274Y	H	H	H	H	H	H	H	H	H
		E276D	E	E	E	E	E	E	E	E	E
		R292K	R	R	R	R	R	R	R	R	R
	Unknown and Mutation was under positive selection	V263I	I	I	V	V	I	V	V	I	I
PB2 ^②^	Increase polymerase activity in avian or mammalian cell line	I66M	M	M	M	M	M	M	M	M	M
		I109V	I	V	I	I	I	I	I	I	I
		I133V	V	V	V	V	V	V	V	V	V
		V255I	V	I	V	V	V	V	V	V	V
		D256G	D	D	D	D	D	D	D	D	D
		T271A	T	T	T	T	T	T	T	T	T
		I292V	I	I	I	I	I	I	I	I	I
		R353K	K	K	R	R	K	K	R	R	R
		K389R	R	R	R	R	R	R	R	R	R
		I495V	V	V	V	V	V	V	V	V	A
		A588V	A	A	A	A	A	A	A	A	A
		V598T/I	T	T	T	T	T	T	T	T	T
		E627K	E	E	E	E	E	E	E	E	E
		D701N	D	D	D	D	D	D	D	D	D
	Identified in H5N8 AIVs and H3N2 CIV canine influenza viruses	V338I	V	I	V	V	V	V	V	V	V
PB1 ^②^	Increased polymerase activity and virulence in mice	N105S	A	A	A	A	A	A	A	A	A
		E198K	K	K	K	K	K	K	K	K	K
		D622G	G	G	G	G	G	G	G	G	G
PA ^②^	Increased polymerase activity in mammalian cell line	S37A	A	A	A	A	A	A	A	A	A
		T97I	T	T	T	T	T	T	T	T	T
		K356R	K	K	K	K	K	K	K	K	K
		N383D	D	D	D	D	D	D	D	D	D
NP ^②^	Increased virulence in mice	V105M	V	M	M	M	V	M	V	I	M
	Increased virulence in chickens	A184K	K	K	K	K	K	K	K	K	K
	Increased polymerase activity in mammalian cell line	N319K	N	N	N	N	N	N	N	N	N
	Increased virulence in mice	I353V	I	V	I	I	V	I	I	I	I
M1 ^②^	Increased virulence in mice	N30D	D	D	D	D	D	D	D	D	D
		T215A	A	A	A	A	A	A	A	A	A
M2 ^②^	Increased resistance to amantadine and rimantadine	L26F	L	L	L	L	L	L	L	L	L
		S31N	S	S	S	S	S	N	S	S	S
NS1 ^②^	Increased virulence in mice	P42S	S	S	S	S	S	S	S	S	S
	Increased virulence in swine and mice	D92E	E	E	E	E	E	E	E	E	E

The amino acid sites of the nine H3N2 subtype AlVs. ^①^ Reference strain: Human influenza A/Aichi/2/1968(H3N2); ^②^ Reference strain: A/Goose/Guangdong/1/1996 (H5N1). Consequently, these results indicated that the LZD44 strain could replicate in multiple tissues in chickens without prior adaptation, exhibiting higher viral titers in multiple tissues than the other two strains did at 3 dpi.

**Table 5:** The glycosylation sites of the NA protein in this study.

Strain	Glycosylation Sites ^①^						
	61–63	69–71	86–88	146–148	200–202	234–236	402–404
NND26	NIT	NNT	NWS	NGT	NAT	NGT	NWS
LZD44	NIT	NNT	NWS	NGT	NAT	NGT	NWS
NND52	NIT	NNT	NWS	NGT	NAT	NGT	NWS
NND54	NIT	NNT	NWS	NGT	NAT	NGT	NWS
NND65	NIT	NNT	NWS	NGT	NAT	NGT	NWS
NND93	NIT	NNT	NWS	NGT	NAT	NGT	NWS
NND98	NIT	NNT	NWS	NGT	NAT	NGT	NWS
NND99	NIT	NNT	NWS	NGT	NAT	NGT	NWS
NND100	NIT	NNT	NWS	NGT	NAT	NGT	NWS

^①^: Location N2 numbering relative to A/Aichi/2/1968 (H3N2).

**Table 6:** The virus titers in multiple tissues of inoculated SPF chickens.

Strains	Time	^①^ The Mean Virus Titers in Multiple Tissues of Inoculated SPF Chickens/log_10_EID_50_/g							
		Brain	Spleen	Kidney	Lung	Liver	Intestines	Trachea	Pancreas
NND98	3 dpi	ND ^②^	ND	ND	3.5 ± 0.25	ND	ND	3.83 ± 0.88	ND
	5 dpi	ND	ND	3.67 ± 0.76	4.42 ± 0.14	2 ± 0.87	2.92 ± 1.38	2.83 ± 0.76	2.25 ± 0.90
NND100	3 dpi	ND	ND	ND	3.67 ± 0.29	ND	ND	3.92 ± 0.38	ND
	5 dpi	ND	ND	ND	2.83 ± 0.29	1.83 ± 0.29	ND	2.5 ± 0.43	ND
LZD44	3 dpi	3.33 ± 0.29 ****	3.5 ± 0.5 **	3.25	7 ± 0.5 *	ND	ND	6.67 ± 1.04 *	3.25 ± 0.75
	5 dpi	1.67 ± 0.29	1.67 ± 0.29	2.17 ± 0.58	4.42 ± 1.01	ND	2.75 ± 1.15	5.42 ± 0.88	4.42 ± 1.01 **
*p* value		*p* < 0.0001	*p* = 0.0031	*p* > 0.05	*p* < 0.05	*p* > 0.05	*p* > 0.05	*p* < 0.05	*p* < 0.01

For statistical analysis, if all three chicken embryos inoculated with tissue homogenate supernatant were HA-negative, the result is recorded as 1.5 log_10_EID_50_/g. ^①^ Mean viral titer: mean ± SD (log_10_EID_50_/g); ^②^ ND: Not detected. Viral titers in the LZD44-inoculated group were compared with those in the NND98- and NND100-inoculated groups by two-way ANOVA with post hoc (Tukey) testing. Statistical significance was represented by: * *p* < 0.05, ** *p* < 0.01, and **** *p* < 0.0001.

**Table 7:** The detection result of viral shedding in oropharyngeal and cloacal swabs of inoculated chickens and contacted chickens.

Strains	Groups	1 dpi		3 dpi		5 dpi		7 dpi		9 dpi		11 dpi		14 dpi	
		T ^①^	C ^②^	T	C	T	C	T	C	T	C	T	C	T	C
NND98	Inoculated ^③^	6/12	2/12	8/12	6/12	9/9	7/9	4/6	4/6	5/6	5/6	6/6	3/6	4/6	0/6
	Contacted ^④^	0/0	0/0	1/3	1/3	3/3	2/3	3/3	2/3	3/3	1/3	3/3	2/3	0/3	0/3
NND100	Inoculated	8/12	1/12	5/12	3/12	7/9	9/9	5/6	4/6	5/6	4/6	6/6	3/6	2/6	1/6
	Contacted	0/0	0/0	0/3	0/3	3/3	3/3	2/3	1/3	3/3	3/3	3/3	1/3	1/3	0/3
LZD44	Inoculated	11/12	0/12	12/12	12/12	9/9	5/9	3/6	1/6	4/6	4/6	2/6	0/6	1/6	0/6
	Contacted	0/0	0/0	3/3	3/3	3/3	3/3	3/3	2/3	2/3	2/3	0/3	2/3	0/3	0/3

The viral shedding in swabs of inoculated and contacted chickens was displayed as: Number of HA-positive swabs/Total number of swabs. ^①^ T: oropharyngeal; ^②^ C: cloacal swab; ^③^ Inoculated: SPF chickens were inoculated with viruses; ^④^ contacted: native chickens that were housed with the inoculated chickens.

**Table 8:** The virus titers in multiple tissues of inoculated mice.

Strain	Time	The Mean Virus Titers in Multiple Tissues of Inoculated Mice ^①^/log_10_EID_50_/g				
		Brain	Spleen	Kidney	Nasal Turbinate	Lung
NND98	3 dpi	2.08 ± 1.01	2.92 ± 1.38	2.67 ± 1.26	2.75 ± 1.09	5.5 ± 2.29
	5 dpi	1.75 ± 0.43	2.5 ± 0.90	2.33 ± 0.76	2.25 ± 0.75	4.25 ± 0.43
NND100	3 dpi	ND ^②^	ND	1.67 ± 0.29	2.33 ± 0.88	5.5 ± 1.32
	5 dpi	1.67 ± 0.29	2.83 ± 1.15	2.67 ± 1.04	1.67 ± 0.29	3.92 ± 0.58
LZD44	3 dpi	1.83 ± 0.58	ND	1.92 ± 0.38	4.17 ± 0.14	7.08 ± 0.72
	5 dpi	2.83 ± 0.29	3.92 ± 0.63	3.92 ± 0.63	4.25 ± 0.75 *	6.67 ± 0.29 *
*p* value		*p* > 0.05	*p* > 0.05	*p* > 0.05	*p* < 0.05	*p* < 0.05

The virus titers in multiple tissues of inoculated mice. For statistical analysis, if all three chicken embryos inoculated with tissue homogenate supernatant were HA-negative, the result is recorded as 1.5 log_10_EID_50_/g. ^①^ Mean viral titer: mean ± SD (log_10_EID_50_/g); ^②^ ND: Not detected. Viral titers in the LZD44-inoculated group were compared with those in the NND98- and NND100-inoculated groups by two-way ANOVA with post hoc (Tukey) testing. Statistical significance was represented by: * *p* < 0.05.

## Data Availability

The data will be accessible upon request, and further inquiries can be directed to the corresponding author.

## Supplementary Materials

The following supporting information can be downloaded at https://www.mdpi.com/article/10.3390/ani16121765/s1: Table S1: The accession numbers of nucleotide sequences of the strains used in this study. Table S2: BLAST analysis of the nine H3N2 subtype AlVs.
