# Supplementary material for: Emergence of Novel Reassortant H3N2 Avian Influenza Viruses in Southern China: Genetic Complexity and Pathogenicity in Chickens and Mice
Source: Animals (Basel). 2026 Jun 8;16(12):1765. doi: 10.3390/ani16121765 (PMC13296187; doi:10.3390/ani16121765)
Supplement: Supplementary file 1 [file animals-16-01765-s001.zip › Supplementary Materials Table S2.pdf]

Table S2. BLAST analysis of the nine H3N2 subtype AIVs.

Gene	Isolates	The strains with highest nucleotide homology retrieved by BLAST[①]	Nucleotide similarity (%)	ORFs /bp
PB2	NND26	A/Bean Goose(Anser fabalis)/Korea/KNU14/2023(H1N1)	99.30	2280 bp
	LZD44	A/duck/Guangzhou/41227/2014(H5N6)	95.17	2280 bp
	NND52	A/duck/China/503D32/2022(H3N2)	99.03	2280 bp
	NND54	A/duck/China/503D32/2022(H3N2)	98.99	2280 bp
	NND65	A/Bean Goose(Anser fabalis)/Korea/KNU14/2023(H1N1)	99.21	2280 bp
	NND93	A/Anseriformes/Anhui/XZ9/2014(H3N8)	93.46	2280 bp
	NND98	A/duck/China/532D55/2023(H3N2)	98.99	2280 bp
	NND99	A/duck/China/532D55/2023(H3N2)	99.21	2280 bp
	NND100	A/duck/China/532D55/2023(H3N2)	99.08	2280 bp
PB1	NND26	A/duck/China/418D46/2020(H4N3)	97.63	2274 bp
	LZD44	A/duck/China/532D55/2023(H3N2)	99.60	2274 bp
	NND52	A/duck/China/532D55/2023(H3N2)	98.28	2274 bp
	NND54	A/duck/China/532D55/2023(H3N2)	98.64	2274 bp
	NND65	A/duck/China/10-31/2022(H6N6)	98.28	2274 bp
	NND93	A/duck/China/532D55/2023(H3N2)	98.20	2274 bp
	NND98	A/duck/China/532D55/2023(H3N2)	99.03	2274 bp
	NND99	A/duck/China/532D55/2023(H3N2)	98.73	2274 bp
	NND100	A/duck/China/532D55/2023(H3N2)	98.72	2274 bp
PA	NND26	A/duck/China/400D17/2019(H3N2)	98.65	2151 bp
	LZD44	A/duck/China/400D17/2019(H3N2)	98.79	2151 bp
	NND52	A/duck/China/400D17/2019(H3N2)	98.88	2151 bp
	NND54	A/duck/China/400D17/2019(H3N2)	98.84	2151 bp
	NND65	A/duck/Guangxi/4130/2020(H3N2)	98.61	2151 bp
	NND93	A/duck/China/503D32/2022(H3N2)<br>A/duck/China/451D28/2021(H4N2)	98.88	2151 bp
	NND98	A/duck/China/400D17/2019(H3N2)	98.65	2151 bp
	NND99	A/duck/China/400D17/2019(H3N2)	99.12	2151 bp

	NND100	A/duck/China/400D17/2019(H3N2)	98.79	2151 bp
HA	NND26	A/chicken/Guangxi/165C7/2014(H3N2)	95.00	1701 bp
	LZD44	A/duck/China/401D23/2019(H3N2)	97.47	1701 bp
	NND52	A/duck/China/401D23/2019(H3N2)	98.18	1701 bp
	NND54	A/duck/China/401D23/2019(H3N2)	98.12	1701 bp
	NND65	A/chicken/Guangxi/165C7/2014(H3N2)	94.77	1701 bp
	NND93	A/duck/Foshan/11/2019(H3N2)	95.30	1701 bp
	NND98	A/chicken/Guangxi/165C7/2014(H3N2)	94.89	1701 bp
	NND99	A/duck/China/403D49/2020(H3N2)	98.53	1701 bp
	NND100	A/duck/China/403D49/2020(H3N2)	98.53	1701 bp
NP	NND26	A/duck/Japan/AQ-HE103/2015(H1N2)	97.33	1495 bp
	LZD44	A/duck/China/330D17/2018(H6N6)	95.59	1495 bp
	NND52	A/duck/China/C2/2022(H6N6)	93.65	1495 bp
	NND54	A/duck/China/532D55/2023(H3N2)	99.06	1495 bp
	NND65	A/Spot-billed duck/South Korea/KNU2020-105/2020(H3N2)	98.86	1495 bp
	NND93	A/duck/China/10-31/2022(H6N6)	99.00	1495 bp
	NND98	A/duck/Japan/AQ-HE103/2015(H1N2)	96.19	1495 bp
	NND99	A/duck/China/532D55/2023(H3N2)	96.06	1495 bp
	NND100	A/duck/China/532D55/2023(H3N2)	99.47	1495 bp
NA	NND26	A/duck/China/272D18/2016(H3N2)	94.82	1410 bp
	LZD44	A/duck/Fujian/06.29FZHX038-O/2021(H2N2)	95.53	1410 bp
	NND52	A/duck/China/532D55/2023(H3N2)	98.44	1410 bp
	NND54	A/duck/China/532D55/2023(H3N2)	98.44	1410 bp
	NND65	A/duck/China/402D22/2019(H3N2)	97.80	1410 bp
	NND93	A/chicken/China/284C1/2017(H3N2)	96.03	1410 bp
	NND98	A/duck/China/272D18/2016(H3N2)	96.67	1410 bp
	NND99	A/chicken/Guangxi/165C7/2014(H3N2)	94.40	1410 bp
	NND100	A/duck/China/402D22/2019(H3N2)	97.30	1410 bp
M	NND26	A/duck/China/10-31/2022(H6N6)	99.19	982 bp
	LZD44	A/pheasant/Hong Kong/SH39/99 (H6N1)	100.00	982 bp

	NND52	A/duck/China/10-31/2022(H6N6)	99.29	982 bp
	NND54	A/duck/China/10-31/2022(H6N6)	99.29	982 bp
	NND65	A/duck/China/10-31/2022(H6N6)	99.39	982 bp
	NND93	A/duck/China/532D55/2023(H3N2)	98.68	982 bp
	NND98	A/duck/China/532D55/2023(H3N2)	99.29	982 bp
	NND99	A/duck/China/532D55/2023(H3N2)	99.39	982 bp
	NND100	A/duck/China/532D55/2023(H3N2)	99.39	844 bp
NS	NND26	A/duck/China/503D32/2022(H3N2)	99.17	844 bp
	LZD44	A/duck/China/503D32/2022(H3N2)	98.46	844 bp
	NND52	A/duck/China/503D32/2022(H3N2)	98.93	844 bp
	NND54	A/duck/China/503D32/2022(H3N2)	98.93	844 bp
	NND65	A/duck/China/503D32/2022(H3N2)	99.17	844 bp
	NND93	A/duck/China/532D55/2023(H3N2)	98.93	844 bp
	NND98	A/duck/China/503D32/2022(H3N2)	99.29	844 bp
	NND99	A/duck/China/503D32/2022(H3N2)	99.29	844 bp
	NND100	A/duck/China/503D32/2022(H3N2)	99.29	844 bp

①: The whole viral genome nucleotide sequences were analyzed for homologous sequences using the BLAST tool provided by the National Center of Biotechnology Information (NCBI).
